# A Clinical Study Comparing the Efficacy of SesameOil with Desensitizing Tooth Paste in Reducing Dentinal Hypersensitivity: A Randomized Controlled Trial

**DOI:** 10.1155/2020/6410102

**Published:** 2020-10-09

**Authors:** Walaa Abdullah Al Qahtani, N. C. Sandeepa, Eman Khalid Abdullah, Yosra Mohammed Mousa, Atheer Abdulhade Ganem, Eman Ali Alqahtani, Afnan Hassan M. Alkhayri

**Affiliations:** King Khalid University, College of Dentistry, Abha, Saudi Arabia

## Abstract

**Objectives:**

The objective of this study was to evaluate the efficacy of sesame oil therapy in reduction of dentin hypersensitivity, as compared to desensitizing dentifrice. *Design, setting, participants*. We conducted a single blinded randomized controlled trial in 100 patients reported to Diagnostic Department of King Khalid University-College of Dentistry between March 2018 and December 2019. *Interventions*. Patients were given desensitizing tooth paste or sesame oil to apply for the specified time. *Main outcome measures*. A Visual Analogue Scale was used to record sensitivity scores for controlled air stimulus and tactile method at the first visit and after 8th week of treatment. Measured outcome was reduction in dentinal hypersensitivity.

**Results:**

Hypersensitivity degree before treatment in case of desensitizing tooth paste was 6.90 ± 1.04, and posttreatment showed a score of 4.70 ± 1.37. In case of sesame oil groups, subjects included had a score of 7.14 ± 0.90 which showed a drop to a score of 4.52 ± 1.16.

**Conclusions:**

Desensitizing tooth paste showed 30.5% reduction in sensitivity, whereas sesame oil application showed 36.2% reduction. The belief of considering oil therapy in oral health is just a placebo effect and may not be considered anymore. Efficacy can be established with many more studies including long follow-up and varying time periods.

## 1. Introduction

Clinical management of dentin hypersensitivity (DH) is highly empirical in spite of a century-old exploration. Although the practice of essential oils is the oldest form of medicine recognized by humankind, their therapeutic uses were still not branded until recently. Many studies have recognized the therapeutic efficacy of essential oils, although they are not incorporated considerably in clinical dentistry.

Theories propose that lipophilic components of edible oils control the bio-adhesion procedure to the teeth and ultrastructure of the initial oral biofilm. A lipid-augmented pellicle might be more impervious in case of acid exposure and could therefore decrease the mineral loss and reduce the symptom of dentin hypersensitivity [1].

Sesame oil was selected for the present research due to its already proven health benefits and easy availability in the household. The sesame plant has been considered a gift of nature. Sesame oil pulling activity is studied in research studies for its effectiveness in dental caries, dental plaque, and halitosis.

The objective of the study was to evaluate the efficacy of sesame oil therapy in reduction of dentin hypersensitivity, as compared to desensitizing dentifrice. This is the first clinical study linking the efficiency of sesame oil with a desensitizing tooth paste in patients with dentinal hypersensitivity.

## 2. Methods

### 2.1. Study Design

The study was approved by the Scientific Research Committee of King Khalid University-College of Dentistry. We randomized patients in a single blinded randomized controlled trial from those reported to diagnostic department and compared the clinical consequences after application of a commonly available desensitizing tooth paste or sesame oil. The study was conducted between March 2018 and December 2019.

### 2.2. Study Population

**Figure 1:**
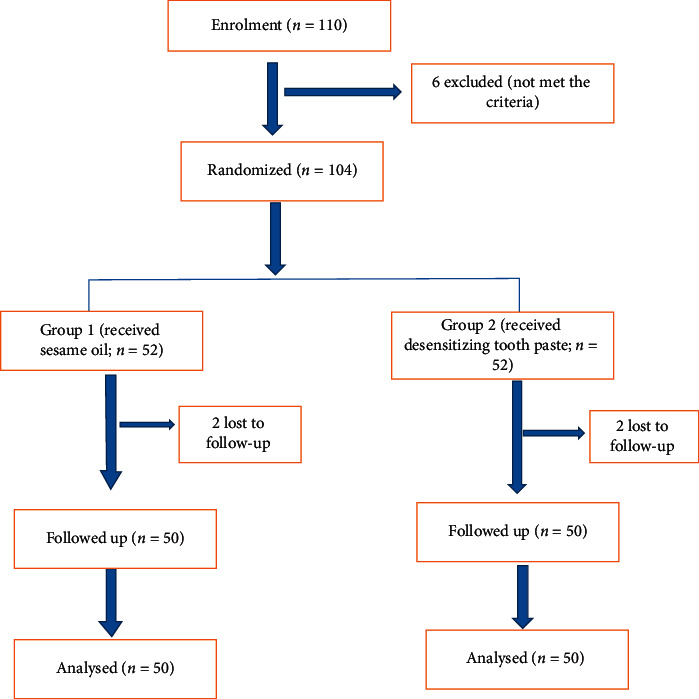
Randomization flow chart.

During the diagnostic procedure, patients were assessed for the sensitivity of teeth. Dentinal hypersensitivity was diagnosed after the proper history and proper assessment methods. Tooth sensitivity was recorded first by the tactile method in which dentin surface was scratched using a sharp probe. Ten minutes following the tactile stimulation, patients' response to cold air was evaluated using a dental air syringe. After completing the entire history and clinical examination, patients were informed about the study protocol and different interventions. By using a simple random method, from the diagnostic department, patients were recruited for the study. Out of 110 patients, six patients were not able to enter the study. Reasons were difficulty to come for the follow-up, travelling to a different place, coming under the exclusion criteria, or not meeting the inclusion criteria. Informed consent was taken from the patient before commencing the study. From 104 patients who entered the study, 52 patients were allotted in each group based on a simple chit system. Two of them lost to follow-up from each group constituting 100 patients in total who completed the study.  Figure 1 describes the randomization procedure followed in the study.

### 2.3. Study Interventions

Group 1 subjects were treated with sesame oil, and Group 2 subjects were given desensitizing tooth paste which contains active ingredients of potassium nitrate and sodium fluoride. As an alternative to oil pulling which can cause certain health issues if not done properly, the method was modified. Patients were told to dab some of the oil with a small piece of cotton wool and apply onto the affected teeth for 10–15 min followed by rinsing the mouth with warm water. Group 2 subjects were advised to rub the prescribed desensitizing dentifrice over the affected surface and leave it for 2 min followed by brushing.

### 2.4. Randomization

Patients were selected randomly from the diagnostic department. Those who were complaining of sensitivity of teeth were assessed based on the proper history and clinical examination. On diagnosing dentinal hypersensitivity, patients were randomly selected based on the simple random method. Every second patient was selected and allotted. Patients were informed about the study. Out of 110 patients, six were not able to enter the study due to various issues of reporting for follow-up or some having the exclusion criteria. Patients who are pregnant or lactating and having sesame oil allergy were excluded from the study. Patients with two or more hypersensitive teeth and having a good general health were included. Patients were distributed into 2 groups based on the simple chit system, either into the tooth paste group or sesame oil group.

### 2.5. Study Outcomes

The primary outcome expected from the study was reduction in the dentinal hypersensitivity. Treatment outcome was calculated using a Visual Analogue Scale which records the sensitivity using a line of length of 10 cm. The line extremes represent the limits of pain a patient might experience from an external stimulus (0 = no pain; 10 = severe pain). Sensitivity scores for controlled air stimulus and tactile method at the beginning and at the 8th week after treatment were documented. Tooth sensitivity was recorded first by the tactile method in which the dentin surface was scratched using a sharp probe. Ten minutes following the tactile stimulation, patients' response to cold air was evaluated using a dental air syringe. The score was documented at the initial visit and at the 8th week, and the result was statistically analyzed.

### 2.6. Sample Size and Statistical Analysis

Sample size analyzed was 100 in number who reported back for follow-up after application of either desensitizing tooth paste or sesame oil. After data collection, data were filtered from any errors, coded, and fed to statistical software IBM SPSS version 21. The constructed graphs were obtained using Microsoft excel software. All statistical analyses were performed using two tailed tests with an alpha error of 0.05. *P* value less than or equal to 0.05 was considered to be statistically significant. Descriptive statistics was used as frequencies, and percent was used to describe the frequency of each category for categorical data. Mean with standard deviation was used to describe quantitative variables (age and hypersensitivity level). The independent *t*-test was used to compare the mean hypersensitivity level between the two groups, while repeated measures with adjusted *P* value was used to compare the sensitivity level before and after intervention.

## 3. Results

### 3.1. Study Population

A total of 100 patients were analyzed at the end of the study. Patients who were less than 30 years were 64%, and 36% of patients were above 30 years. A total of 85 females and 15 males participated in the study.  Table 1 shows demographic characteristics of the sampled cases according to intervention type regarding hypersensitivity. The sesame oil group comprised 46 females (92%) and 4 males (8%), while the tooth paste group had 39 females (78%) and 11 males (22%).  Table 2 shows comparison of tooth hypersensitivity degree between the two study groups before and after applying the interventions. Hypersensitivity degree before treatment in case of desensitizing tooth paste was 6.90 ± 1.04, and posttreatment showed a score of 4.70 ± 1.37. In case of sesame oil groups, subjects included had a score of 7.14 ± 0.90 which showed a drop to a score of 4.52 ± 1.16.

Desensitizing tooth paste showed 30.5% reduction in sensitivity, whereas sesame oil application showed 36.2% reduction. Percentage of reduction was calculated by subtracting preintervention level from postintervention level multiplied by 100 and then dividing by preintervention level ((posttreatment score − pretreatment score) × 100 ÷ pretreatment score).

### 3.2. Major Complications

None of the patients had presented with any serious adverse effects or complication in the two-month follow-up. A few subjects reported discomfort due to taste of the oil.

## 4. Discussion

DH is a painful clinical condition with an incidence ranging from 4 to 74%. The variations in the reports may be because of dissimilarity in populations and different investigation approaches [2, 3]. It is reported more frequently in females [4, 5]. The employed methods to diagnose DH in various research studies were patient questionnaires or clinical examinations [2, 3].

There are numerous treatment modalities accessible for DH which can be either performed at home or may be professionally applied. The usual “at home” desensitizing agents include toothpastes, mouthwashes, or chewing gums which act by either hindering the neural transmission or sealing the dentinal tubules. There are some recent treatment choices such as bioglass, Portland cement, lasers, and casein phosphopeptides [6]. Alternatively, homeopathic medications including Plantago major and propolis are also seen as effective modalities [7].

According to the World Oral Health report, despite great advances in dental health, the dental health problems still remain and are predominantly observed among underprivileged groups in both developing and developed countries [8]. In this context, many traditionally used medicines for treating infections have been considered again, due to the emerging problems related to overuse of antibiotics. Clinical trials are being performed to establish their efficacy and possible side effects. One of these categories of natural medicines is essential oils (EOs). In the current years, there has been an amplified attention towards EOs [9].

Many people including healthcare providers are not conscious of benefits of essential oils. In recent years, due to adverse effect of available routine drugs, consumers are in search of natural medicine for their healthcare requirements. This has led to increased use of essential oils which are readily available and part of our daily life. Essential oils are very concentrated in such a way that, sometimes, the whole plant is used to yield a drop of oil [10].

The sesame plant has been considered as a gift of nature. Sesame root contains chlorosesamone which attributes to its antifungal action [8]. Sesame oil is rich in polyunsaturated fatty acids which can lessen free radical injury happening in the oral cavity [11]. Sesame oil is a rich source of sesamin, sesamolin, and sesaminol which play a vital role in detoxification. It has antioxidant and antibiotic activities and also deters lipid peroxidation [12].

An in vitro study by Asokan et al. in 2011 observed that health effects of sesame oil on oral health are due to saponification, emulsification, and mechanical cleansing [13]. Traditionally, sesame oil is considered to be the ideal one for oil pulling [13]. Studies have revealed that sesame oil and sunflower oil has an important role in reducing plaque-induced gingivitis [14].

This is the first clinical study to find the therapeutic effect of sesame oil in the management of DH as compared to desensitizing tooth paste. Instead of oil pulling therapy, which is the routine method of treatment of oral conditions, the method of application was modified to local application over the affected area.

Sunflower oil was studied in dentinal hypersensitivity by Cheema et al. in 2014 [1]. Oil pulling therapy was used instead of local application. Oil pulling was performed in the following way. On an empty stomach, 10 ml of sunflower oil was sipped, sucked, and swished for 20–25 minutes till oil lost its viscosity and turned milky white in colour and then was spit out, and the mouth was rinsed with water. It was observed that there was reduction in VAS scores in both cold-pressed sunflower oil and desensitizing tooth paste groups. However, statistically significant reduction in the mean VAS score of DH was detected in the group who performed oil pulling [1].

In another study, thyme oil which is an essential oil was used for the management of dentinal hypersensitivity [15]. It was found that both desensitizing tooth paste and thyme oil was effective in managing DH. On comparing, it was found that thyme oil was effective in reducing moderate pain/hypersensitivity than desensitizing tooth paste (*P* < 0.001). No association was detected for the sensitivity score of anterior or posterior teeth with pretreatment and posttreatment of both intervention groups [15].

Our result with sesame oil showed that there was a reduction in sensitivity in both the groups, 30.5% decrease in case of desensitizing tooth paste subjects and 36.2% in case of sesame oil application. It shows the effectiveness of oil and can be used as a good alternative treatment option which is readily available in households and as a cost-effective intervention. Cost of sesame oil for 8-week application was less than the cost of tooth paste required for application.

Toxicological checks are often deficient for traditional medicines. Therefore, further clinical trials are necessary to eliminate the likelihood of side effect. In our study, there were no adverse effects reported except discomfort regarding the taste of the oil. If used properly, traditional medicines may ascertain very beneficial in dental therapy and may contribute in improving the quality of dental treatments.

## 5. Conclusion

Essential oils are not a replacement in routine dental care, but it can be considered as an alternative noninvasive method when employed properly. As research continue to arise on the benefits of oils in oral health, the practice of them may become a common place in dental care. Dental practitioners can feel assured with the research facts so as to recommend or support a patient's demand to use oils. When it comes to a traditional medicine, the main component which can be missing is the toxicological tests. Therefore, further clinical trials are essential to eliminate the likelihood of side effect. Extensive research on the role of traditional, cheap, and valuable remedy should be encouraged without bias.

## Figures and Tables

**Table 1:** Demographic characteristics of the sampled cases according to intervention type regarding hypersensitivity.

Demographic characteristics of sampled cases	Type			
	Sesame oil		Tooth paste		*P*
	No	%	No	%	
*Age in years*					
Less than 30 years	23	46.0%	41	82.0%	0.001^*∗*^
More than 30 years	27	54.0%	9	18.0%	

Mean ± SD	31.7 ± 8.9		26.8 ± 8.7		

*Gender*					
Female	46	92.0%	39	78.0%	0.053
Male	4	8.0%	11	22.0%	

**Table 2:** Comparing tooth hypersensitivity degree between the two study groups before and after applying the interventions.

Phase	Type					
	Sesame oil			Tooth paste			*P* ^$^
	Range	Mean	SD	Range	Mean	SD	
Hypersensitivity degree before	5–9	7.14	.90	5–9	6.90	1.04	0.202
Hypersensitivity degree after	2–7	4.52	1.16	2–7	4.70	1.37	0.481

*P* ^#^	0.001^*∗*^			001^*∗*^			

## Data Availability

Data will be made available upon contacting Dr Walaa Abdullah Al Qahtani via email (drwala3333@gmail.com).
